# Integrons and multidrug resistance across phylogenetic groups of clinical isolates of *Escherichia coli*

**DOI:** 10.12669/pjms.40.6.8886

**Published:** 2024-07

**Authors:** Rimsha Razzaq, Ahmad Sheraz, Muhammad Mohsin Arshad, Asad Bashir Awan, Abdul Haque

**Affiliations:** 1 Rimsha Razzaq Department of Health Biotechnology, Akhuwat FIRST, Faisalabad, Pakistan; 2 Ahmad Sheraz Department of Health Biotechnology, Akhuwat FIRST, Faisalabad, Pakistan; 3 Muhammad Mohsin Arshad Department of Health Biotechnology, Akhuwat FIRST, Faisalabad, Pakistan; 4 Asad Bashir Awan Department of Health Biotechnology, Akhuwat FIRST, Faisalabad, Pakistan; 5 Abdul Haque Department of Health Biotechnology, Akhuwat FIRST, Faisalabad, Pakistan

**Keywords:** *Escherichia coli*, Multidrug resistance, Extensively drug resistance, Integrons, Phylogenetics groups, Multiplex PCR

## Abstract

**Objective:**

This study was aimed to investigate the multidrug resistance patterns in clinical isolates of *Escherichia coli* and their correlation with integrons and phylogenetic groupings.

**Methods:**

A total of 37 clinical *E. coli* isolates were evaluated for drug resistance patterns by disk diffusion method. Phylogenetic groupings and the presence of integrons among *E. coli* were determined by multiplex PCR assays.

**Results:**

Multidrug resistance was identified in 84% of the clinical isolates of *E. coli* with higher resistance found against cephalosporins (94.6%) and fluoroquinolones (83.8%), while lower resistance was observed against polymyxins (24.3%) and carbapenems (29.7%). Metallo-β-lactamases were found in all carbapenem resistant isolates. The phylogenetic group B2 was the most dominant (40.5%), followed by groups A (35.1%), D (13.5%) and B1 (10.8%). Integrons were detected in 25 (67.6%) isolates and *intI1*, *intI2*, and *intI3* genes were found in 62.2%, 18.9% and 10.8% of isolates respectively.

**Conclusion:**

Our results show that phylogenetic classification of *E. coli* is not relevant with antimicrobial resistance. However, there was strong association between the integron classes and resistance against β-lactam and fluoroquinolones antimicrobials. Additionally, this study highlighted that the presence of integrons plays a crucial role in the development of multidrug resistance in clinical isolates of *E. coli*. Most significantly, this is the first report of detection of three classes of integron among clinical isolates of *E. coli* in Pakistan.

## INTRODUCTION

*Escherichia coli* (*E. coli*) is a widely studied bacterial species, serving as a prototype for a diverse range of strains that can be both commensal and pathogenic. While some *E. coli* strains are benign and have no harmful effects on the host, others can cause a variety of diseases, including enteritis, diarrhea, urinary tract infections, septicaemia, and neonatal meningitis, both in humans and other animal hosts.^1^ The majority of *E. coli* strains live in the gastrointestinal tracts of humans and animals as commensals without causing any harm. However, certain strains of *E. coli* have evolved to become dangerous by acquiring virulence factors through transposons, plasmids, pathogenicity islands and bacteriophages. These virulence factors allow the pathogenic *E. coli* strains to cause infections and diseases, which can have severe consequences for the hosts.^2^

Antimicrobial resistance in pathogenic microorganisms is a critical global health issue that has emerged in recent times. Pathogenic microorganisms are classified into four categories based on their resistance to antimicrobials: pandrug-resistant, extensively-drug-resistant (XDR), multidrug-resistant (MDR), or non-multidrug-resistant. This categorization is based on whether they are resistant to all, most, or just a few antibiotic classes.^3^

The classification of *E. coli* strains is done into four main phylogenetic groups, namely A, B1, B2, and D. The majority of commensal *E. coli* strains fall under group A and B1. However, the most virulent extra-intestinal strains primarily belong to group B2 and, to a lesser extent, group D. The molecular typing is done through PCR amplification based on the genetic markers such as *ChuA*, which is a gene essential for heme transport in Enterohemorrhagic O157:H7 *E. coli*; *yjaA*, a gene coding a protein (uncharacterized protein); and *TspE4*.*C2*, a putative DNA fragment.^4^

Integrons were originally characterized by Hall and Collis in 1989 as genetic elements with the capability to capture and express genes. Within the 50 conserved portion of this element (PC), there are three crucial components: an integrase gene (*IntI*) responsible for encoding a site-specific recombinase enzyme, an *attI* site, recognized by the integrase and uses as a receptor for gene cassettes, and a promoter region. When integrated, the gene cassettes are added to the integron. Although integrons are not mobile per se, their mobility is conferred by genetic elements like transposons or plasmids that can move from one bacterium to another. Therefore, integrons represent a significant mechanism facilitating the dissemination of multidrug resistance.^5^

Until recently there are five classes of integrons based on the nucleotide sequence of the integrase gene. The Class-I integrons, in comparison to Class-II and 3 integrons, pose a major threat to human health by spreading antimicrobial resistance (AMR) against major antimicrobial groups due to relatively higher abundance, distribution and mobility.^6^ The Class-II integrons have been commonly reported in some species of Gram-negative organisms such as *Acinetobacter*, *Enterobacteriaceae*, *Salmonella* and *Pseudomonas*. This class is mainly associated with aminoglycosides resistance. The class three integrons are relatively least abundant, however, they are associated with resistance against tetracycline, β-lactams, aminoglycosides, and anti-metabolites antimicrobials.^7^ The class-4 and 5 integrons have been associated with *Vibrio cholera* and *Alivibrio salmonicida*.^8^ This study was designed to segregate local clinical *E. coli* isolates on the basis of drug resistance, phylogenetic groups and integron distribution; and to find correlations, if any.

## METHODS

### Collection of specimens:

The isolates of *Escherichia coli* were obtained from Microbial Strains Collection Bank of Akhuwat-Faisalabad Institute of Research Science and Technology (FIRST), Pakistan. These isolates were previously collected from human blood, urine, sputum, pus, throat, and ears samples from Madina Teaching Hospital, Faisalabad, Pakistan in 2019.^9^

### Ethical Approval:

This study was approved by the Institutional Ethical Committee of Akhuwat-FIRST, Faisalabad with reference number: Akt-FIRST/P21/ethics/05, dated February 23, 2021.

### Phenotypic identification of isolates:

The stock cultures were enriched in TSB (Tryptic Soy Broth) and then inoculated onto MacConkey agar (Merck) plates. Characteristic colonies indicating lactose fermentation were isolated and subjected to further screening via standard biochemical tests including MR-VP (Methyl Red - Voges-Proskauer) and TSI (Triple Sugar Iron). After phenotypic identification of *E. coli* isolates, glycerol stocks of bacterial cultures were prepared (25% v/v glycerol in TSB) and stored at -80°C for future use.

### PCR based confirmation of E. coli:

PCR was used to validate the presence of *E. coli* isolates by amplifying the *uid A* gene responsible for encoding β-glucuronidase, which is a specific marker for *E. coli* detection. The forward and reverse primers employed were 5’-ATCACCGTGGTGACGCATGTCGC-3’ and 5’-CACCACGATGCCATGTTCATCTGC-3’, respectively, with a product size of 486 bp.^10^

### PCR based phylogenetic analysis:

Each isolate was classified into one of the four major phylogenetic groups (A, B1, B2, and D) using a triplex PCR that targeted the DNA fragments *TspE4*.*C2*, *chuA*, and *yjaA* as markers.^4^ The primer sequences and product sizes are given in Table-I.

**Table-I:** List of primers along with respective amplicon sizes, used for phylogenetic groupings and integrons detection.

Gene	Primer sequence		Product size
***Phylogenetic***			
chuA	F	GACGAACCAACGGTCAGGAT	279 bp
	R	TGCCGCCAGTACCAAAGACA	
yjaA	F	TGAAGTGTCAGGAGACGCTG	211 bp
	R	ATGGAGAATGCGTTCCTCAAC	
TspE4.C2	F	GAGTAATGTCGGGGCATTCA	152 bp
	R	CGCGCCAACAAAGTATTACG
***Integrons***			
intI1	F	ATCATCGTCGTAGAGACGTCGG	893 bp
	R	GTCAAGGTTCTGGACCAGTTGC	
intI2	F	GCAAATGAAGTGCAACGC	467 bp
	R	ACACGCTTGCTAACGAT	
intI3	F	GCAGGGTGTGGACAGATACG	760 bp
	R	ACAGACCGAGAAGGCTTA	

### PCR based detection of integrons:

Primers designed to target the integrase genes (*intI1*, *intI2*, and *intI3*) were employed in a multiplex PCR assay to identify Class-I, Class-II, and class 3 integrons in multidrug-resistant (MDR) *E. coli* isolates.^11^,^12^ The primer sequences and product sizes are given in Table-I.

### PCR and thermocycler conditions:

In 50 μl reaction mixture of multiplex PCR for both phylogenetic analysis and detection of integrons, the final concentrations of components were as follows: 1×Taq buffer, 1.5 mM MgCl2, 0.2 mM dNTPs mixture, 0.4 μM of each primer, two units of Taq polymerase, and PCR water to achieve the desired volume. The DNA template (5 μl) was taken from a 25 ng/μl stock solution. The thermal cycler conditions consisted of an initial denaturation step at 94°C for five minutes, followed by 30 cycles of denaturation at 94°C for one minute, annealing at 55°C for one minute, and extension at 72°C for one minute. Finally, there was a concluding extension step at 72°C for 10 minutes. To visualize the amplicons, electrophoresis was performed by loading the PCR products onto a 1.5% agarose gel.^4^

### Antimicrobial susceptibility testing:

In adherence to the guidelines outlined by the Clinical & Laboratory Standards Institute (CLSI, 2020) and the European Committee on Antimicrobial Susceptibility Testing (EUCAST breakpoints v.12.0, 2022), the antimicrobial susceptibility testing was performed using the disk diffusion method. The disks of antimicrobial agents utilized in this assessment were Cefoxitin (30μg), Levofloxacin (5μg), Ceftazidime (30μg), Amikacin (30μg), Cefotixime (30μg), Ciprofloxacin (5μg), Amoxicillin-CA (30μg), and Doripenem (10μg). Metallo-β-lactamases were also phenotypically detected using previously reported methodology.^13^ *E. coli* ATCC 25922 was used as quality control for the antimicrobials.

### Statistical analysis:

The Google Sheets™ were used for data recording, analysis, and graphical representations. “CHITEST” function was used to assess the significant associations between antimicrobial resistance and the presence of the integron classes.

## RESULTS

### Phenotypic and molecular identification of E. coli isolates:

All *E. coli* isolates showed semi-dome shaped, non-mucoid and pink color colonies on MacConkey agar plates. Species specific PCR based upon *uidA* gene amplification confirmed the identification of all 37 *E. coli* isolates.

### Antimicrobial susceptibility testing:

The antimicrobial drug resistance patterns exhibited by these isolates are shown in Table-II. The majority of the isolates were resistant to cephalosporins [94.6%] and fluoroquinolones [83.8%]. Additionally, more than 50% isolates were resistant to augmentin (amoxicillin-clavulanic acid), nitrofurantoin and aminoglycosides. According to international criteria for multidrug resistance patterns, MDR isolates are resistant to at least one member of three or more antimicrobial groups while XDR isolates are resistant to at least one member of all antimicrobial groups with exception of maximum two antimicrobial groups.^14^ Out of 37 isolates, 17 [46%] were identified as MDR and 14 [38%] were identified as XDR isolates with a total of 84% isolates exhibiting multidrug resistance patterns. Overall, the isolates exhibited least resistance to polymyxins [24.3%] and carbapenems [29.7%] respectively. Metallo-β-lactamase activity was detected among all carbapenem resistant isolates.

**Table-II:** Comparison of resistant and sensitive isolates against each antimicrobial groups.

Antimicrobials	Resistant isolates		Sensitive isolates	

	(n)	(%)	(n)	(%)
Augmentin	22	59.5	15	40.5
Cephalosporins	35	94.6	2	5.4
Carbapenem	11	29.7	26	70.3
Fluoroquinolones	31	83.8	6	16.2
Aminoglycosides	19	51.4	18	48.6
Nitrofurantoin	21	56.8	16	43.2
Polymyxins	9	24.3	28	75.7

### Phylogenetic analysis:

**Fig. 1:**
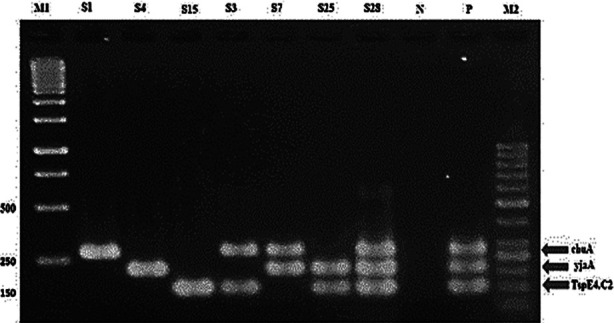
Triplex PCR profiles specific for E. coli phylogenetic groups. Lane 1:M1=GeneRuler™ 1Kb ladder (Thermo Scientific, Cat # SM0313); Lane 2 contains group D; Lane 3 contains group A; Lane 4 and 5 contains group B1; Lane 6 contains group D; Lane 7 and 8 contains group B2; Lane 9 contains negative control; Lane 10 contains positive control; Lane 11:M2= GeneRuler™ 50bp ladder (Thermo Scientific, Cat # SM0373).

Among 37 isolates, 15 (40.5%) and 13 (35.1%) isolates belonged to the phylogenetic groups B2 and A respectively. While, 5 (13.5%) and 4 (10.8%) isolates belonged to the D and B1 phylogenetic groups respectively. A representative gel picture for the triplex PCR for the phylogenetic typing is given in Fig.1.

The distribution of different phylogenetic groups in terms of multidrug resistance patterns is given in Table-III. Interestingly, no significant difference (*p*-value= 0.699) was found among phylogenetic groups of A, B2, B1 and D (Fig. 3A).

**Table-III:** Multidrug resistance profiles of E. coli isolates based on phylogenetic groupings.

Isolates	A	B1	B2	D
Non-MDR (Non-multidrug resistant)	0	0	4 (26.7%)	2 (40%)
MDR (Multidrug resistant)	7 (53.8%)	2 (50%)	7 (46.7%)	1 (20%)
XDR (Extensively drug resistant)	6 (46.2%)	2 (50%)	4 (26.7%)	2 (40%)

**Fig. 2:**
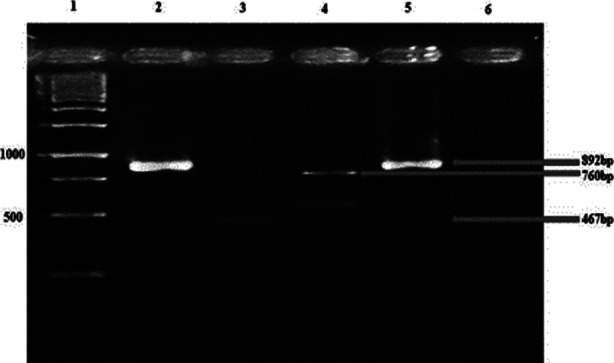
Multiplex PCR for integrons. Lane 1:M=GeneRuler™ 1 Kb ladder (Thermo Scientific, Cat # SM0313); Lane 2: intI1 (892bp); Lane 3:intI 2 (467bp): Lane 4:intI 3: (760bp); Lane 5: intI1 (upper band =892bp) and intI2 (lower band=467 bp); Lane 5: negative control PCR (PCR mix without template DNA).

**Fig.3:**
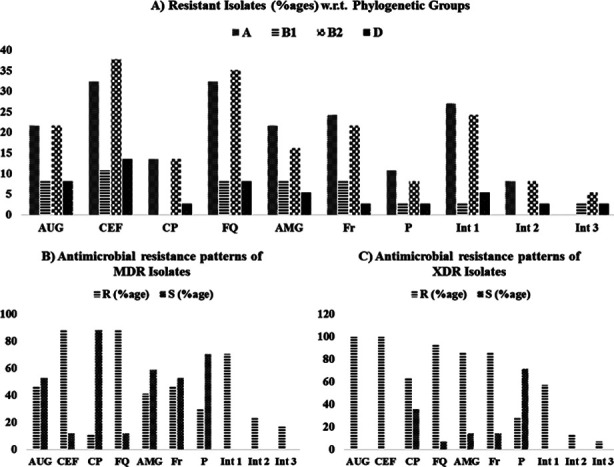
Distribution of resistant isolates in percentages **A)** among respective phylogenetic groups **B)** among MDR (multi-drug-resistant) isolates and **C)** among XDR (extensively-drug-resistant) isolates. Int=integron class; AUG=amoxicillin-clavulanic acid; CEF=cephalosporins; CP = Carbapenem; FQ = Fluoroquinolones; AMG =aminoglycosides; Fr=furans; P=polymyxins; R=resistant; S= sensitive.

### Detection of integrons:

Integrons, concomitantly present in some isolates, were detected in a total of 25 (67.6%) isolates. The majority of detected integrons [22 (62.2%) isolates] belonged to Class-1. The Class-II and 3 integrons were observed in 7 (18.9%) and 4 (10.8%) isolates respectively. The concomitant presence of Class-I and Class-II as well as Class-I and Class- 3 was detected in four isolates each. However, Class-II and class 3 were not concomitantly found in any of the isolates.

The presence of integrons was significantly associated (*p*-value: <0.05) with antimicrobial resistance against cephalosporins (*p*-value: 0.00001) and fluoroquinolones (*p*-value: 0.00001) as per the applied chi-square statistical test. However, in case of Augmentin, only Class-II and class-3 integrons exhibited significant association (*p*-value: 0.00001). Meanwhile, no significant differences were observed in case of aminoglycosides (*p*-value: 0.60) and nitrofurantoin (*p*-value: 0.86). Contrary to that, integrons were found significantly more in sensitive isolates than in resistant isolates for carbapenems (*p*-value: 0.009) and polymyxins (*p*-value: 0.009) These associations can also be visualized in Fig.3 in terms of multidrug resistance patterns.

## DISCUSSION

*E. coli* is a major pathogen causing a range of diseases due to its versatility. It is also one of the most deeply studied pathogens in the world. However, the molecular characteristics of clinical isolates of *E. coli* in relation to antimicrobial resistance has not been comprehensively studied covering all geographical locations of Pakistan. A major mechanism of distribution of drug resistance genes is via integrons that can identify, integrate, and express antimicrobial resistance-coding gene cassettes in clinical *E. coli* isolates.^15^

The presence of integrons has been previously associated with β-lactam resistance genes.^7^ The antimicrobial resistance against Augmentin in this study was less as compared to above-mentioned studies, indicating that the products of β-lactamase genes carried by integrons were effectively inhibited by clavulanic acid. In addition, we found that carbapenem was relatively more effective than other β-lactam antimicrobials. This observation is at variance with a previous report that associated carbapenem resistance with integrons.^16^ This might be due to either absence of genes or additional mechanisms such as efflux pumps related to carbapenem resistance or innately weaker promoter resulting in weaker expression.^17^ The antimicrobial resistance against fluoroquinolones was also strongly associated with the presence of integrons in our study. Many studies have reported this association previously in terms of presence of plasmid-mediated quinolones resistance (PMQR) genes, however, they could not detect any resistance gene directly from the integrons.^18^

In our study, it was important to note that all the four isolates harboring class 3 integrons were all MDR or XDR isolates. Even in the latest “One Health” studies, the class 3 integrons have rarely been found among Gram negative bacteria.^5^,^19^ However, this class of integrons has been detected in clinical isolates of *Proteus mirabilis* and *E. coli* in Egypt and Iran respectively.^19^,^20^ In another report from France, Class- 3 integrons were detected in the fecal matter but not directly in Gram negative bacteria.^21^ In Pakistan, only one study from KP [Khyber Pakhtunkhwa] region reported the detection of class 3 integrons in *E. coli* isolates from poultry retail meat.^22^ In our study, this class was not detected in phylogenetic Group- A as shown in Fig.3A. Furthermore, the simultaneous occurrence of two types of integrons in one *E. coli* isolate, which was rarely observed in the past, has recently been observed in lower frequencies in clinical *E. coli* isolates.^23^

Majority of the isolates showed higher resistance against cephalosporin and fluoroquinolone as compared to previous reports.^24^ However, the combination amoxicillin-clavulanic acid was found relatively more effective due to β-lactamase inhibition. This scenario has been reported in earlier studies from Pakistan.^25^ In our study, for non-MDR isolates, amoxicillin-clavulanic acid remained the most effective antimicrobial. However, with the presence of carbapenemases (metallo-β-lactamases) among MDR and XDR isolates, the isolates were found resistant to all β-lactam antimicrobials including carbapenems, cephalosporins and penicillin even with clavulanic acid as β-lactamase inhibitors. This is in line with previous findings from various studies.^26^ In addition to that, aminoglycosides and nitrofurantoin were found less effective against XDR isolates (as shown in Fig.3C) which is in line with previous studies.^27^,^28^ Carbapenems were found as the most effective antimicrobials followed by polymyxins against MDR isolates (Fig. 3B) while for XDR isolates only polymyxins were the only effective antimicrobials (Fig. 3C). Similar findings for XDR isolates have been previous reported in which polymyxins were recommended either as a single ingredient or in combination with other antimicrobial groups.^29^,^30^

The phylogenetic group has been reported to play an important role in predicting the pathogenicity and virulence of the pathogens. In most of the studies, the B2 phylogenetic group has been reported as the most common isolates in extraintestinal infections.^31^ In the present study, frequency of isolations from B2 (40.5%) and A (35.1%) phylogenetic groups was similar. This finding is in agreement with some other studies.^32^ It might be due to horizontal gene transfer allowing the commensal *E. coli* (Group A and Group B1) strains to acquire several virulence factors facilitating their infections in the urinary tract, in addition to opportunistic diarrheal infections.^33^

### Limitations of the study:

The findings may not represent the diversity of *E. coli* strains in different clinical settings at different geographical locations.

## CONCLUSION

In conclusion, the presence of integrons was associated with antimicrobial resistance against β-lactams (excluding carbapenems) and fluoroquinolones. Polymyxins and carbapenems were the most effective antimicrobials against *E. coli* isolates. The presence of MDR and XDR isolates in the otherwise commensal isolates from groups A and B1 represents an alarming public health situation in the management of urinary tract and gastrointestinal infections. This is the first report from Pakistan where three classes of integrons were detected in the clinical isolates of *E. coli*.

### Authors’ Contribution:

**RR:** Disk diffusion assays, PCR.

**AS:** Biochemical and phenotypic characterization, PCR.

**MMA:** Sample revival and data analysis.

**ABA:** Experimental validation, troubleshooting and manuscript drafting.

**AH:** Concept and overall supervision. Responsible and accountable for the accuracy or integrity of the work.
